# The Effects of Palmitoylethanolamide or Ibuprofen on the Abundance Profile and Synthesis Rate of Proteins in C2C12 Skeletal Myotubes

**DOI:** 10.1096/fba.2025-00286

**Published:** 2026-04-23

**Authors:** Paige L. Cole, Connor A. Stead, Jatin G. Burniston, Daniel J. Owens

**Affiliations:** ^1^ Research Institute of Sport and Exercise Sciences Liverpool John Moores University Liverpool UK

**Keywords:** deuterium oxide, non‐steroidal anti‐inflammatory drugs, protein turnover, proteomics, skeletal muscle

## Abstract

Palmitoylethanolamide (PEA) and ibuprofen (IBU) exert anti‐inflammatory effects that may influence skeletal muscle adaptation; however, their impact on muscle proteome dynamics remains unclear. Dynamic proteome profiling was performed in differentiated C2C12 myotubes treated for 36 h with D_2_O and either vehicle control (VC), PEA (10 μM), or IBU (100 μM). Protein‐specific fractional synthesis rates (FSR; 1541 proteins) and relative protein abundances at 36 h (3085 proteins) were quantified and compared between treatments and VC. Relative to VC, PEA increased synthesis rates of 101 proteins (*p* < 0.05), whereas 2 proteins exhibited reduced synthesis. IBU increased synthesis rates of 165 proteins and reduced 7 proteins relative to VC. Both PEA and IBU increased total ribosomal protein synthesis (~80% relative to VC) and increased the abundance of 40S ribosomal subunit proteins (~18% relative to VC at 36 h). In addition, IBU treatment was associated with greater abundance of proteins involved in muscle contraction and extracellular matrix organization, reduced abundance of proteins associated with carbohydrate metabolism (21 proteins), and increased abundance of proteins linked to lipid metabolic pathways (17 proteins), relative to VC. In contrast, PEA‐induced abundance differences were largely restricted to ribosomal proteins. These findings demonstrate that PEA and IBU enhance ribosomal protein turnover relative to control, whereas IBU elicits broader treatment‐associated proteome remodeling.

## Graphical abstract

(a) C2C12 myoblasts were cultured and differentiated over 7 days until mature myotubes were formed. (b) Myotubes were treated with PEA (10 μM), IBU (100 μM) or VC for 36 h. Simultaneously, cells were incubated with D_2_O (deuterium oxide) for isotopic labeling. Protein was harvested at 12 and 36 h post‐treatment and analyzed using (c) liquid chromatography‐mass spectrometry (LC–MS) to quantify de novo protein synthesis (12–36 h labeling period) and relative protein abundance at the 36 h time point. (d) Statistical and bioinformatic analysis was then performed on 1541 protein‐specific fractional synthetic rates and 3085 protein abundances.

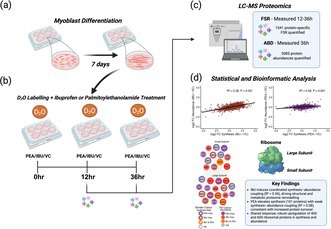

## 1 Introduction

Palmitoylethanolamide (PEA), a naturally occurring fatty acid amide, has recently been identified as a promising alternative to non‐steroidal anti‐inflammatory drugs (NSAIDS) due to its analgesic and anti‐inflammatory properties [1, 2]. PEA acts through mechanisms distinct from NSAIDs, notably by modulating proliferator‐activated receptor alpha (PPARα) and G protein‐coupled receptor 55 (GPR55) [3]. In addition, PEA has been reported to indirectly target transient receptor potential vanilloid receptor 1 (TRPV1) [4] and CB_1_ and CB_2_ receptors [5]. Unlike NSAIDS, PEA does not inhibit cyclooxygenase or prostaglandin synthesis, suggesting it may attenuate inflammation and pain without interfering with pathways involved in muscle protein synthesis (MPS). PEA has beneficial effects in models of neuropathic pain [6], neuroinflammation [7], and tissue injury [8], yet its influence on skeletal muscle recovery and adaptive signaling pathways following exercise‐induced muscle damage (EIMD) is unexplored.

Whilst PEA has not been explored in the context of EIMD, NSAIDs such as ibuprofen (IBU) are routinely used in both recreational and elite sports, primarily to manage pain and inflammation associated with EIMD. Surveys conducted on ultra‐endurance runners indicate that NSAID use is high and is often undertaken without medical supervision [9, 10]. The adverse effects of chronic NSAID use are well‐established, including a heightened risk of upper gastrointestinal issues [11] and shorter‐term NSAID use during training and competition to alleviate EIMD‐related symptoms is prevalent amongst endurance sports. Despite widespread use and growing awareness of potential adverse effects, few studies have evaluated how NSAIDs may influence muscle adaptive responses and cellular processes at the molecular level.

The effects of NSAIDs on human muscle adaptation may be context‐dependent. In older individuals, daily consumption of IBU (1200 mg·day^−1^) or acetaminophen (ACET; 4000 mg·day^−1^) during a 12‐week programme of progressive resistance training did not impair muscle mass gains, and in some individuals, protentiated muscle hypertrophy and gains in strength [12]. Similarly, a moderate dose of IBU (400 mg·day^−1^) had no detrimental effect on hypertrophy in young individuals [13], whereas a high dose of IBU (1200 mg·day^−1^) attenuated muscle growth and strength gains [14]. The effects of local infusion of NSAIDs on human muscle adaptation are also unclear. In one study, NSAID infusion inhibited satellite cell responses to resistance exercise [15], whereas a separate study using an identical infusion protocol found no impact on muscle protein synthesis or gene expression following eccentric exercise [16]. These discrepancies across prior studies may reflect underlying differences in age‐related inflammatory profiles and training status, highlighting the complexity of interactions between NSAIDs and muscle responses to exercise.

Acute studies have provided further mechanistic insight by demonstrating that NSAID consumption can blunt the exercise‐induced increase in muscle protein synthesis following resistance exercise, possibly through reduced prostaglandin signaling. Trappe et al. [12] reported that skeletal muscle fractional synthesis rate (FSR), measured 24 h post‐exercise, was significantly increased in the placebo group but remained unchanged in the IBU and ACET group. This may be partly explained by the observation that NSAID administration attenuates the exercise‐induced increase in prostaglandin F_2_α (PGF_2_α) and prostaglandin E_2_ (PGE_2_) protein levels, lipid mediators known to regulate protein turnover [17, 18]. Earlier in vitro and animal studies showed that prostaglandins released from contracting muscle exert complex effects on protein turnover, stimulating synthesis via PGF_2_α and influencing degradation pathways via PGE_2_ [19, 20, 21]. However, these studies relied on radioisotope labeling and measured mixed‐protein synthesis, limiting the resolution of protein‐specific responses. As a result, the exact mechanism of action of NSAIDs on anabolic and catabolic pathways remains unclear. Dynamic proteome profiling offers a more powerful approach by combining stable isotope labeling with peptide mass spectrometry to quantify protein turnover and abundance at the individual level [22]. We have previously used dynamic proteome profiling to illustrate how specific proteins and pathways are remodeled during C2C12 differentiation [23]. Such resolution is critical for determining whether NSAID treatment impairs or selectively alters these processes.

To address this, the aims of this work were twofold: first, to evaluate the muscle protein synthetic responses to either IBU or PEA treatment using dynamic proteome profiling, assessing both bulk fractional synthesis rates (FSR) and protein‐specific FSR in response to each treatment. Second, to assess proteome remodeling by integrating protein‐specific FSR with protein abundance. By integrating the measurement of protein‐specific abundance and synthesis rates the effects of IBU and PEA on muscle proteome dynamics can be captured in an unbiased manner. Through direct application of these treatments to differentiated myotubes in vitro, this study aimed to provide the first comprehensive analysis of the effects of IBU and/or PEA treatment on the synthesis of muscle proteins assessed on a protein‐by‐protein basis. Furthermore, we provide the first direct investigation of IBU or PEA on muscle cells to understand whether PEA represents a potential, mechanistically distinct alternative to NSAIDs, capable of attenuating inflammation without compromising anabolic signaling or protein synthesis pathways involved in muscle adaptation and recovery.

## 2 Materials and Methods

### 2.1 Chemicals, Reagents, and Plasticware

PEA (molecular mass 299.5 g/mol) was obtained from Sigma Aldrich (product number P0359; Sigma Aldrich, Gillingham, UK) in powdered form and reconstituted in ethanol to 50 mg/mL (≈167 mM). IBU (molecular mass 206.29 g/mol) was purchased from Sigma Aldrich (product number I4883; Sigma Aldrich, Gillingham, UK) in powdered form and reconstituted in ethanol to 50 mg/mL (≈243 mM). Stocks of both substances were stored at −20°C and used within 3 weeks and were diluted in culture media as required for experiments.

Dulbecco's modified eagle medium (DMEM; Gibco, product number 11995073), fetal bovine serum (FBS; Gibco, product number A5256701), new‐born calf serum (NBCS; Gibco, product number 26010074), penicillin–streptomycin (pen‐strep.; Gibco, product number 15070063), trypsin–EDTA (Gibco, product number 25300054), and horse serum (HS; Gibco, product number 26050088) were purchased from Thermo Fisher Scientific (Oxford, UK). Phosphate buffered saline (PBS; Sigma‐Aldrich, product number P4417) tablets, DMSO (Sigma‐Aldrich, product number 472301), and gelatin from porcine skin (Sigma‐Aldrich, product number G9136) were purchased from Sigma‐Aldrich (Gillingham, UK). T75 culture flasks and 6‐well culture plates (Nunc, product number 150239) were purchased from Thermo Fisher Scientific, Oxford, UK. C2C12 myoblasts were purchased from ATCC (LGC Standards, Middlesex, UK). All experiments detailed in this manuscript were performed on C2C12s between passage 5 and 10.

### 2.2 Cell Culture

C2C12 murine myoblasts were cultured in gelatin (0.2%) coated 6‐well culture plates in humidified 5% CO_2_ at 37°C in growth media comprising DMEM, 10% FBS, 10% NBCS and 1% pen‐strep solution. Upon reaching ~80% confluence, monolayers were washed twice with pre‐warmed PBS and cultured in low serum differentiation media (DM), DMEM, 2% HS and 1% pen‐strep. Every 48 h thereafter, DM was removed from monolayers via aspiration and was replaced with fresh media for 7 days. Isotopic labeling of newly synthesized proteins was achieved by supplementing media with 4% deuterium oxide (D_2_O). To investigate protein abundance and FSR during late differentiation, separate cell cultures were incubated in DM containing either H_2_O or D_2_O during 7–10 days of differentiation. Proteins extracted from control cells (DM + H_2_O) were used to measure the natural isotopic abundance of proteins in the absence of D_2_O.

### 2.3 Cell Treatments

Pilot experiments reported in Cole et al. [24], investigated C2C12 myotube viability across a range of PEA (1, 10, and 100 μM) identifying 10 μM PEA as the optimal tolerable dose for subsequent experiments. For ibuprofen (1, 10, and 100 μM), concentrations were selected to reflect commonly used in vitro ranges. Based on previous literature demonstrating biological efficacy with acceptable viability at this dose in skeletal muscle cell models, 100 μM ibuprofen was selected for subsequent experiments [25, 26]. Given their distinct molecular targets and mechanisms of action, the study was not designed to directly compare PEA and IBU against each other, nor to determine relative potency between compounds. Rather, each treatment was evaluated relative to vehicle control to characterize compound‐specific effects on proteome dynamics. Accordingly, statistical analyses were structured as treatment–control comparisons rather than direct PEA versus IBU contrasts (see Section 2.8).

**FIGURE 1:**
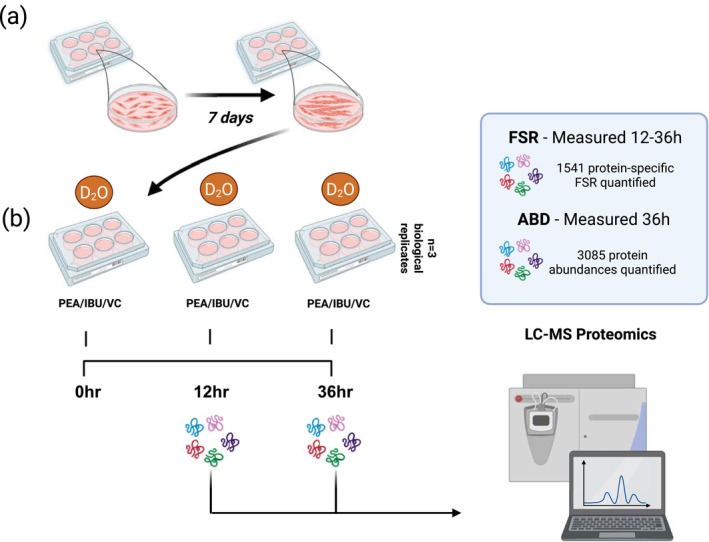
Experimental design for assessing protein synthesis and abundance in C2C12 myotubes. (a) C2C12 myoblasts were cultured and differentiated over 7 days until mature myotubes were formed. (b) Myotubes were treated with PEA (10 μM), IBU (100 μM), or VC for 36 h. Simultaneously, cells were incubated with D_2_O (deuterium oxide) for isotopic labeling. Protein was harvested at 12 and 36 h post‐treatment and analyzed using liquid chromatography‐mass spectrometry (LC–MS) to quantify de novo protein synthesis (12–36 h labeling period) and relative protein abundance at the 36 h time point. Fractional synthesis rates (FSR) were calculated based on deuterium incorporation between 12 and 36 h, whereas changes in relative protein abundance (ABD) were determined from total protein levels at 36 h.

On Day 7 of differentiation, differentiation medium (DM) was aspirated, monolayers were washed twice with PBS and treated with fresh DM containing either vehicle control (DMSO), PEA (10 μM) or IBU (100 μM). All treatments (vehicle control, PEA, and Ibu) were applied in parallel within the same experimental run to minimize inter‐experimental variability. For each independent experiment, treatment allocation to wells was randomized across plates. Plates were positioned randomly within the incubator and rotated between shelves where applicable to minimize positional bias related to temperature, humidity, or CO_2_ gradients. All media changes and treatment applications were performed using a standardized handling procedure, with identical volumes, timing, and incubation durations across conditions. Treatments were prepared fresh on the day of application and added sequentially in a balanced order to avoid systematic timing differences between groups. Myotubes were cultured in their respective treatments for 36 h, with protein collected at two time points (12 and 36 h) for the analysis of FSR and protein abundance (see Figure 1).

### 2.4 Proteomic Analysis

Cells were washed twice with ice‐cold PBS, prior to incubation on ice in 250 μL/well RIPA buffer (0.5 M Tris–HCL, pH 7.4, 1.5 M NaCI, 2.5% deoxycholic acid, 10% NP‐40, 10 mM EDTA) including cOmplete protease inhibitor cocktail (Roche, product number 11697498001; Basel, Switzerland) for 5 min. Thereafter, cells were harvested using a cell scraper and stored at −80°C. Protein concentrations of each protein sample were measured using the Bradford assay (product number B6916; Sigma‐Aldrich, Poole, Dorset, United Kingdom) against bovine serum albumin (BSA) standards (0–2 mg·mL^−1^) prepared in RIPA buffer.

Muscle proteins were processed for mass spectrometry by tryptic digestion according to the filter aided sample preparation (FASP) method [27]. Lysates containing 100 μg protein were precipitated in 5 volumes acetone at −20°C for 1 h. After centrifugation (5000× *g*, 5 min), acetone was decanted and the protein pellets were air dried and then resuspended in 200 μL of UA buffer (8 M urea, 100 mM tris, pH 8.5). Samples were incubated at 37°C for 15 min in UA buffer with 100 mM dithiothreitol (DTT) followed by 20 min at 4°C in UA buffer containing 50 mM iodoacetamide (protected from light). Samples were washed twice with 100 μL UA buffer and transferred to 50 mM ammonium hydrogen bicarbonate (Ambic). Sequencing grade trypsin (Promega, Madison, WI, USA) in 50 mM Ambic was added at an enzyme to protein ratio of 1:50 and the samples were digested overnight at 37°C. To terminate digestion, peptides were collected in 50 mM Ambic and trifluoroacetic acid (TFA) was added to a final concentration of 0.2% (v/v). Aliquots, containing 4 μg peptides, were desalted using C_18_ Zip‐tips (Millipore, Billerica, MA, USA) and resuspended in 0.1% formic acid spiked with 10 fmol μL^−1^ yeast alcohol dehydrogenase (ADH1) (Waters Corp., Milford, MA) in preparation for liquid chromatography‐mass spectrometry (LC–MS/MS) analysis.

### 2.5 Liquid Chromatography–Tandem Mass Spectrometry

Peptide mixtures were analyzed using an Ultimate 3000 RSLC nano liquid chromatography system (Thermo Scientific) coupled to Q‐Exactive orbitrap mass spectrometer (Thermo Scientific). Samples were loaded on to the trapping column (Thermo Scientific, PepMap Neo, 5 μm C18, 300 μm × 5 mm), using ulPickUp injection, for 1 min at a flow rate of 25 μL/min with 0.1% (v/v) TFA and 2% (v/v) ACN. Samples were resolved on a 110 cm analytical column (μPAC Neo; Thermo Fisher) using a gradient of 97.5% A (0.1% formic acid) 2.5% B (79.9% ACN, 20% water, 0.1% formic acid) to 50% A: 50% B over 150 min at a flow rate of 300 nL/min. Data‐dependent selection of the top‐10 precursors selected from a mass range of *m*/*z* 300–1600 was used for data acquisition, which consisted of a 70,000‐resolution full‐scan MS scan at *m*/*z* 200 (AGC set to 3e6 ions with a maximum fill time of 240 ms). MS/MS data were acquired using quadrupole ion selection with a 3.0 *m*/*z* window, HCD fragmentation with a normalized collision energy of 30 and in the orbitrap analyzer at 17,500‐resolution at *m*/*z* 200 (AGC target 5e4 ion with a maximum fill time of 80 ms). To avoid repeated selection of peptides for MS/MS, the program used a 30 s dynamic exclusion window. The mass spectrometry proteomics data have been deposited to the ProteomeXchange Consortium via the PRIDE [28] partner repository with the dataset identifier PXD069882 and https://doi.org/10.6019/PXD069882.

### 2.6 Label‐Free Quantification of Protein Abundance

Progenesis Quantitative Informatics for Proteomics (QI‐P; Nonlinear Dynamics, Waters Corp., Newcastle, UK) was used for label‐free quantitation (LFQ), consistent with previous studies [23, 29]. MS data were normalized by inter‐sample abundance ratio, and relative protein abundances were calculated using nonconflicting peptides only. MS/MS spectra were exported in Mascot generic format and searched against the Swiss‐Prot database (2022_08) restricted to “Rattus” (8071 sequences) using locally implemented Mascot server (v.2.8 www.matrixscience.com) with automatic target‐decoy search. The enzyme specificity was trypsin with 2 allowed missed cleavages, carbamidomethylation of cysteine (fixed modification) and oxidation of methionine (variable modification). *M*/*Z* error tolerances of 10 ppm for peptide ions and 20 ppm for fragment ion spectra were used. The Mascot output (xml format), restricted to non‐homologous protein identifications was recombined with MS profile data in Progenesis. Protein abundance data were calculated using only unique peptides with identification scores of < 1% false‐discovery rate (FDR).

### 2.7 Dynamic Proteome Profiling

Mass isotopomer abundance data were extracted from MS spectra using Progenesis Quantitative Informatics (Non‐Linear Dynamics, Newcastle, UK). Consistent with previous work [23, 29], the abundances of peptide mass isotopomers were collected over the entire chromatographic peak for each proteotypic peptide that was used for label‐free quantitation of protein abundances. Mass isotopomer information was processed using in‐house scripts written in Python (version 3.12.4). The incorporation of deuterium into newly synthesized protein was assessed by measuring the increase in the relative isotopomer abundance (RIA) of the m1 mass isotopomer relative to the sum of the m0 and m1 mass isotopomers that exhibits rise‐to‐plateau kinetics of an exponential regression [30] as a consequence of biosynthetic labeling of proteins in vivo.

(1) $$\mathrm{RIA}=\frac{m_{1}}{m_{0}+m_{1}}$$

The plateau in RIA (RIA_plateau_) of each peptide was derived from the total number (*N*) of 2H exchangeable H–C bonds in each peptide, which was referenced from standard tables [31], and the difference in the D:H ratio (2H/1H) between the natural environment (DH_nat_) and the experimental environment (DH_exp_) based on the molar percent enrichment of deuterium in the precursor pool, according to [32].

(2) $${\mathrm{RIA}}_{\text{plateau}}=1-\left(\frac{1}{\left(\frac{1}{1-\mathrm{RIA}\left(t_{0}\right)}\right)+N\left({\mathrm{DH}}_{\exp}-{\mathrm{DH}}_{\mathrm{nat}}\right)}\right)$$

The rate constant of protein degradation (*k*_deg_) was calculated between the beginning (*t*_0_) and end (*t*_1_) of each 36‐h labeling period. Calculations for exponential regression (rise‐to‐plateau) kinetics reported in [32] were used and *k*_deg_ data were adjusted for differences in protein abundance (*P*) between the beginning (*t*_0_) and end (*t*_1_) of each labeling period.

(3) $$k_{\deg}=-\frac{1}{t-t_{0}}\cdot \ln\left(1-\frac{\mathrm{RIA}\left(t_{1}\right)-\mathrm{RIA}\left(t_{0}\right)}{\;{\mathrm{RIA}}_{\text{plateau}}-\mathrm{RIA}\left(t_{0}\right)}\;\right)\cdot \frac{\;P\left(t\right)}{P\left(t_{0}\right)}$$

Fractional synthesis rates (FSR) for individual peptides were derived by multiplying peptide *k*_deg_ by 100. Individual protein FSR were calculated as the median FSR of unique peptides comprising the parent protein.

### 2.8 Statistical and Bioinformatic Analysis

Unless stated otherwise, data are presented as mean ± standard deviation (SD) and statistical analyses were conducted in R (version 4.5.1). All proteomic data were filtered to exclude proteins that were not quantified across all conditions (VC, IBU, and PEA) in all replicates (*n* = 3 biological replicates (independent culture plates), in each group) to avoid reliance of data imputation. Protein abundance was initially explored across all three conditions (VC, IBU, PEA) at 12 and 36 h using a two‐way ANOVA with condition and timepoint as factors with summaries of these results are provided in Data S1 and Figure S1. For the primary discovery analysis, given the aim of independently cataloguing the proteomic response of each compound relative to a common baseline rather than directly comparing mechanistically distinct treatments, statistical comparisons were performed as independent one‐way ANOVAs for each treatment against VC separately (ibuprofen vs. VC and PEA vs. VC). Protein abundance was evaluated at 36 h and protein fractional synthesis rates were assessed across the 12–36 h labeling window. For individual protein abundance and synthesis data, Type I error was controlled by calculating false discovery rates (*q* values) using the “*q* value” package (R/Bioconductor) [33], reported alongside *p* values throughout. Proteins exhibiting a statistically significant effect of condition (*p* < 0.05) on abundance or synthesis rate were taken forward for downstream bioinformatic analysis.

To assess global changes in protein synthesis rates the median fractional synthesis rate was calculated in each replicate and compared between conditions. The similarity between protein‐specific synthesis responses to IBU and PEA treatment was assessed by Pearson's correlation, comparing log_2_ fold changes (treatment/control) in fractional synthesis rate between conditions. Correlation analysis was conducted using Pearson's correlation to assess the association between protein‐specific log_2_ fold changes in fractional synthesis rate and abundance within each treatment, to provide an estimate of the relationship between changes in synthesis and proteome remodeling.

Functional enrichment of significantly up‐ and down‐regulated proteins was performed using the clusterProfiler package [34] with the org.Mm.eg.db database. Gene Ontology (GO), biological process (BP), molecular function (MF), and cellular component (CC) terms were tested using the enrichGO function, with adjusted *p*‐values calculated using the Benjamini–Hochberg method. The top 10 most significantly enriched terms were reported and visualized.

For specific analysis of ribosomal proteome responses proteins containing the term “ribosome” in their description were extracted from the full dataset and classified as cytosolic or mitochondrial based on subunit annotation (40S/60S or 28S/39S, respectively). Within the cytosolic subset, proteins were further grouped by subunit (40S or 60S), and median FSR and total abundance were calculated per replicate and treatment condition to provide representative subunit‐level measures of ribosomal protein dynamics. Data were analyzed using one‐way analysis of variance (ANOVA) to assess the effect of treatment (ibuprofen and PEA vs. vehicle control). Where a significant main effect of treatment was identified (*p* < 0.05), pairwise comparisons were performed using Tukey's post hoc test to determine differences between treatment and control conditions.

To visualize networks of proteins of interest, bibliometric mining in the Search Tool for the Retrieval of INteracting Genes/proteins using the evidence of interaction sources, experimental verified protein–protein interaction data, GO databases, and co‐expression data with the minimum required interaction score set at 0.4 (medium confidence) were used (STRING, version 12). Protein–Protein interaction networks were visualized using the STRINGdb package in R (version 2.12.1) [35]. Protein–protein interaction networks were transferred via the RCy4 R packaged (version 1) and visualized using Cytoscape version 3.9.1 [36].

To assess the relationship between changes in protein synthesis and steady‐state abundance, log_2_ fold changes (treatment vs. vehicle control) were calculated for each protein. Pearson's correlation analysis was then performed to determine the degree of association between synthesis rate and abundance responses within each treatment condition offering insight into the role of changes in degradation on proteome remodeling.

## 3 Results

### 3.1 Dynamic Proteome Profiling of C2C12 Myotubes

Dynamic proteome profiling was conducted on proteins that had high‐quality peptide mass isotopomer profiles. In total, we quantified the fractional synthesis rates (FSR; %/h) and abundance (femtomole/μg protein; fmol/μg) of 2513 and 3328 proteins, respectively. Prior to statistical analyses, all data were stringently filtered to exclude proteins that were not quantified in all conditions (VC, IBU, and PEA) across all replicates (*n* = 3 in each group). After filtering, 1541 protein‐specific FSR were quantified across the 12–36 h measurement period in all conditions and replicates. Furthermore, 3085 protein abundances, quantified after 36 h of VC, IBU, or PEA treatments, were used for statistical analysis.

### 3.2 Mixed Protein Fractional Synthesis Rates

**FIGURE 2:**
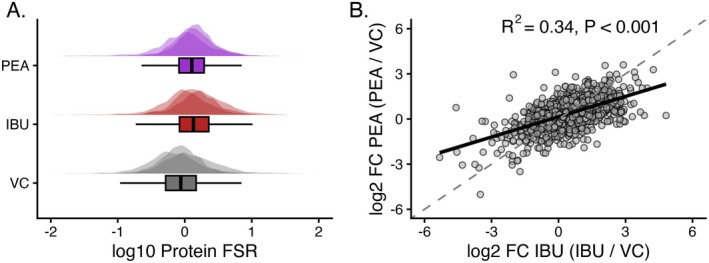
Global differences in fractional synthesis rates (FSR) across experimental conditions. Density plots illustrate the distribution of 1541 proteins (log_2_‐transformed FSR values; %/h) across each sample replicates (*n* = 3) for each condition: Vehicle control (VC, gray), palmitoylethanolamide (PEA, blue), and ibuprofen (IBU, red). Boxplots represent condition‐level summaries of global protein synthesis rates. (B) Scatterplot visualizing differences in the changes of protein‐specific FSR (*n* = 1541 proteins) between IBU and PEA conditions. Points represent mean log_2_ fold change in FSR relative to vehicle control (IBU/VC or PEA/VC). Pearson correlation revealed condition‐specific changes in proteins between IBU and PEA, overall highlighting that whilst global patterns of changes in FSR may appear similar between conditions (A) the individual proteins responding in FSR are largely different between conditions (B).

Global changes in protein synthesis rates were assessed by calculating mixed‐protein FSR for each sample (median FSR of 1541 individual protein FSR). One‐way ANOVA revealed no significant difference (*p* = 0.184) in the mixed protein FSR between VC (0.88% ± 0.25%/h), IBU (1.37% ± 0.38%/h), and PEA (1.27% ± 0.24%/h) (Figure 2A). Differences in protein synthetic responses (log_2_ fold change treatment/control) showed limited concordance between IBU and PEA treatments (*R* ^2^ = 0.34, *p* < 0.001), indicating that while the overall direction of response was similar, the treatments induced distinct protein‐specific alterations (Figure 2B).

### 3.3 Alterations in Proteome Dynamics in Response to Ibuprofen

**FIGURE 3:**
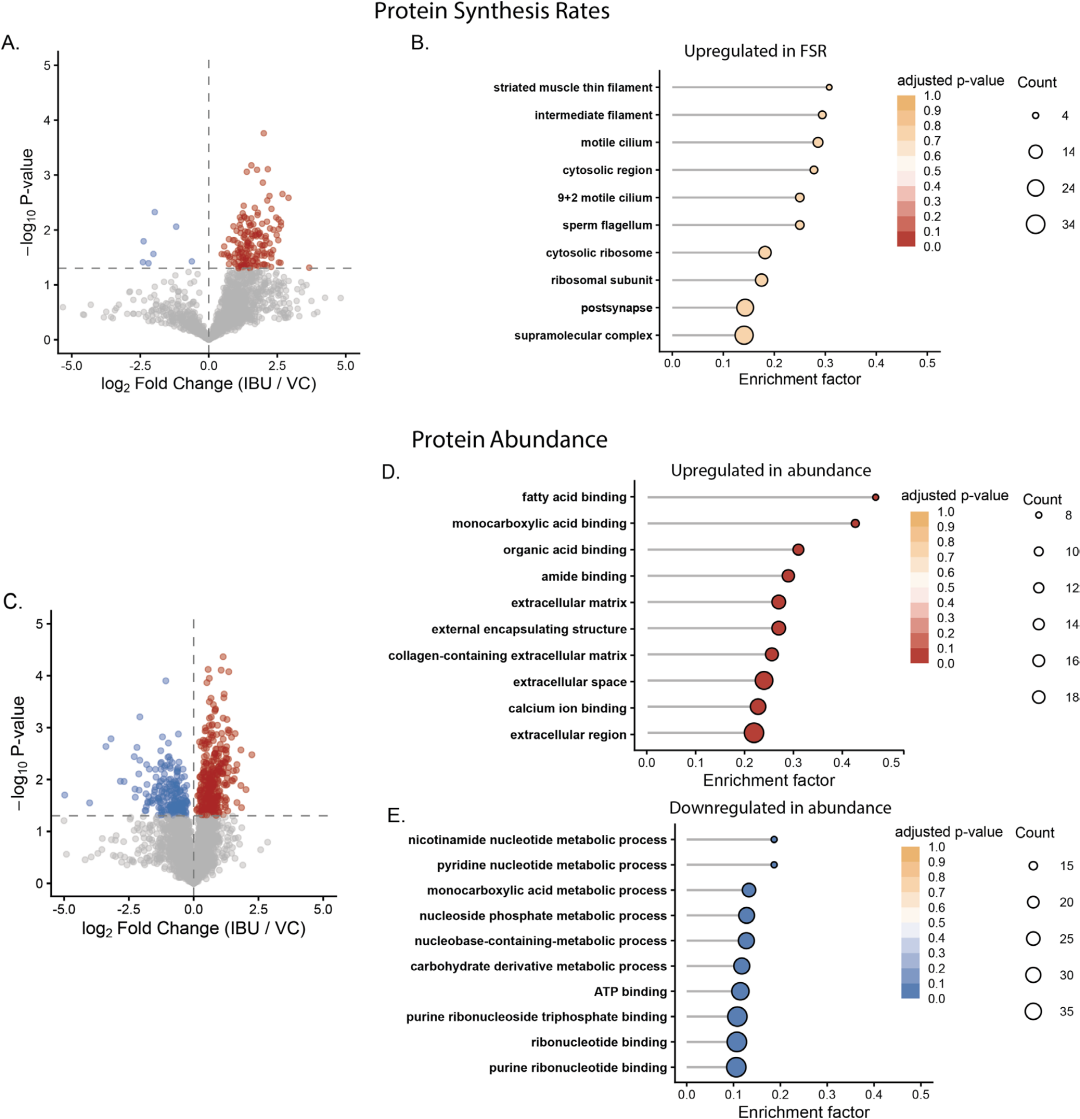
Changes in muscle proteome dynamics in response to ibuprofen treatment. (A) Volcano plot showing protein‐specific changes in fractional synthesis rate (FSR; log_2_ fold change IBU/VC, *x*‐axis) and corresponding –log_10_ *p*‐values (*y*‐axis). Points represent individual proteins detected across conditions; colors denote significantly (*p* < 0.05) upregulated (red) and downregulated (blue) proteins relative to vehicle control (VC). (B) Gene Ontology (GO) enrichment analysis of proteins with increased FSR following ibuprofen treatment. (C) Volcano plot showing protein abundance changes (log_2_ fold change IBU/VC). (D, E) GO enrichment analyses for proteins with increased (D) or decreased (E) abundance. GO terms reflect the top 10 enriched terms ranked by *p*‐value. Point size indicates the number of proteins in each GO term, and color intensity reflects the adjusted *p*‐value.

Protein‐specific FSR were quantified across the 12–36 h treatment period and compared using a one‐way ANOVA to assess changes in FSR between IBU and VC conditions on a protein‐by‐protein basis. A total of 172 proteins exhibited significant (*p* ≤ 0.05, *q* < 0.18) differences in FSR. Amongst the 172 proteins exhibiting significant differences, > 95% (165) of proteins increased in synthesis rate in IBU treated cells. Functional enrichment analysis revealed no clear over‐representation of specific GO terms amongst the proteins upregulated in synthesis rate by IBU treatment. Rather, the most enriched terms (adjusted *p* = 0.79) were associated with generic terms attributed to muscle cell development such as “supramolecular complex” (39 proteins), “ribosomal subunit” (14 proteins; Rpl19, Rpl22, Rpl28, Rpl3, Rpl35, Rpl35a, Rpl38, Rplp2, Rps14, Rps19, Rps20, Rps21, Rps27a, and Fau), and “striated muscle thin filament” (4 proteins; Actn3, Tnnt2, Tpm1, and Tpm2) (see Figure 3B). Therefore, indicating IBU induced a global increase in protein synthesis rates across numerous subclasses of proteins.

To assess the extent of proteome remodeling following IBU or PEA treatment, protein abundances were initially evaluated across all three conditions (IBU, PEA, and VC) at 12 and 36 h timepoints using a two‐way ANOVA with condition and timepoint as factors (Figure S1; Data S1). Four hundred eighteen proteins demonstrated a nominal treatment‐by‐time interaction (*p* < 0.05), reflecting proteins whose temporal trajectory differed across conditions. Hierarchical clustering of these proteins resolved five clusters (Figure S1), dominated by two opposing ibuprofen‐specific patterns: Cluster 1 (*n* = 229) captured proteins selectively increased by ibuprofen between 12 and 36 h which included networks of proteins associated with mitochondria, muscle contraction and structure, and alternative splicing, whereas Cluster 5 (*n* = 173) captured proteins reciprocally suppressed over the same period. Together accounting for 96% of all interaction‐significant proteins.

To independently catalogue the proteomic response of each compound relative to a common baseline, subsequent discovery analyses were performed as independent one‐way ANOVAs comparing each treatment against VC at 36 h separately, as detailed in the methods.

Following 36 h of IBU treatment, a total of 561 proteins were significantly altered (*p* ≤ 0.05, *q* < 0.16), with 352 proteins increased and 209 decreased in abundance following IBU treatment. In contrast to the global anabolic protein synthetic response, proteins increased in abundance were enriched for GO terms associated with extracellular matrix remodeling (e.g., extracellular matrix; 21 proteins and collagen‐containing extracellular matrix; 20 proteins), as well as calcium ion binding (31 proteins; adjusted *p* = 0.03), amide binding (18 proteins; adjusted *p* = 0.03), monocarboxylic acid binding (9 proteins; adjusted *p* = 0.03), and fatty acid binding (8 proteins; adjusted *p* = 0.03). Large networks of proteins associated with the GO terms “myofibril” (26 proteins; adjusted *p* = 0.17) and “ribosome” (19 proteins; adjusted *p* = 0.23) were also increased in response to IBU treatment (Figure 3D).

From the 208 proteins decreased in abundance following IBU treatment, functional enrichment analysis identified enrichment of GO terms associated with ribonucleotide binding (50 proteins; adjusted *p* = 0.03) and carbohydrate metabolic processes (21 proteins; adjusted *p* = 0.07), including the regulatory subunit (gamma‐1) of 5′‐AMP‐activated protein kinase (AMPK), hexokinase‐2, the pyruvate dehydrogenase (PHD) inhibitor—PDH kinase isozyme 2 and 3 isoenzymes of phosphofructokinase (PFKAL and PFKAP, respectively) (see Figure 3E). Thus, indicating IBU treatment increased the abundance of proteins associated with muscle growth and structural development alongside re‐wiring of metabolic pathways through increased abundance of proteins associated with lipid metabolism and decreased abundance of proteins associated with carbohydrate metabolism.

### 3.4 Alterations in Protein‐Specific FSR and Abundance During Treatment With PEA

**FIGURE 4:**
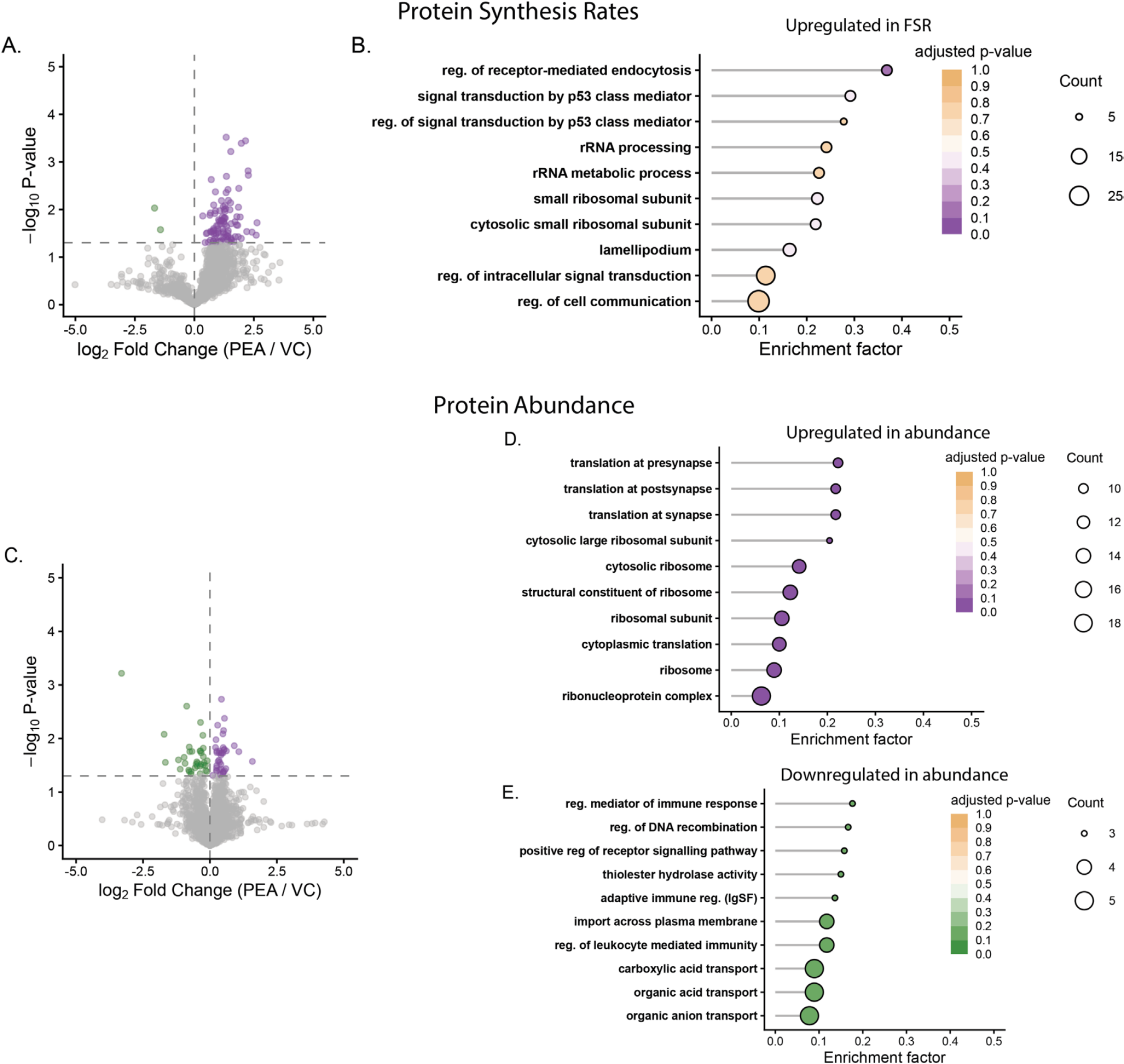
Changes in muscle proteome dynamics in response to PEA treatment. (A) Volcano plot showing protein‐specific changes in fractional synthesis rate (FSR; log_2_ fold change PEA/VC, *x*‐axis) and corresponding –log_10_ *p*‐values (*y*‐axis). Points represent individual proteins detected across conditions; colors denote significantly (*p* < 0.05) upregulated (purple) and downregulated (green) proteins relative to vehicle control (VC). (B) Gene Ontology (GO) enrichment analysis of proteins with increased FSR following PEA treatment. (C) Volcano plot showing protein abundance changes (log_2_ fold change PEA/VC). (D, E) GO enrichment analyses for proteins with increased (D) or decreased (E) abundance. GO terms reflect the top 10 enriched terms ranked by *p*‐value. Point size indicates the number of proteins in each GO term, and color intensity reflects the adjusted *p*‐value.

Protein‐specific FSR were quantified across the 12–36 h treatment period and compared using a one‐way ANOVA to assess changes in FSR between PEA and VC conditions on a protein‐by‐protein basis. A total of 103 proteins exhibited significant (*p* ≤ 0.05, *q* < 0.43) differences in FSR. In line with the response to IBU, 101/103 proteins were increased in synthesis rate in response to PEA treatment. A number of the top 10 enriched GO terms were associated with the ribosome and in particular a network of eight proteins associated with the 40S small ribosomal subunit (Npm1, Rps14, Rps16, Rps2, Rps20, Rps27l, Rps3, Rps8) were significantly increased in synthesis rate in response to PEA treatment (see Figure 4B). Furthermore, from a total of 75 proteins significantly (*p* ≤ 0.05, *q* < 0.99) changed in protein abundance following PEA treatment, GO terms associated with translation and the ribosome were significantly enriched (adjusted *p* < 0.001) within the 39 proteins upregulated in PEA treated cells. These included 14 proteins comprising the ribosome (Rpl10, Rpl13a, Rpl22, Rpl28, Rpl3, Rpl35, Rpl35a, Rpl36, Rpl7, Mrpl28, Rps14, Rps20, Rps27, and Rps3). Therefore, highlighting PEA results in similar global increases in protein synthetic responses, however, results in much more targeted and coordinated proteome remodeling primarily focussed on upregulation of ribosomal proteins (see Figure 4D).

In contrast GO terms associated with the 36 proteins decreased in abundance following PEA treatment highlighted a downregulation of proteins associated with regulation of the immune response (Abcd3, Slc12a2, Slc15a4, Slc27a4, Septin2) (see Figure 4E).

### 3.5 Ibuprofen and PEA Increase Ribosomal Protein Abundance and Turnover

**FIGURE 5:**
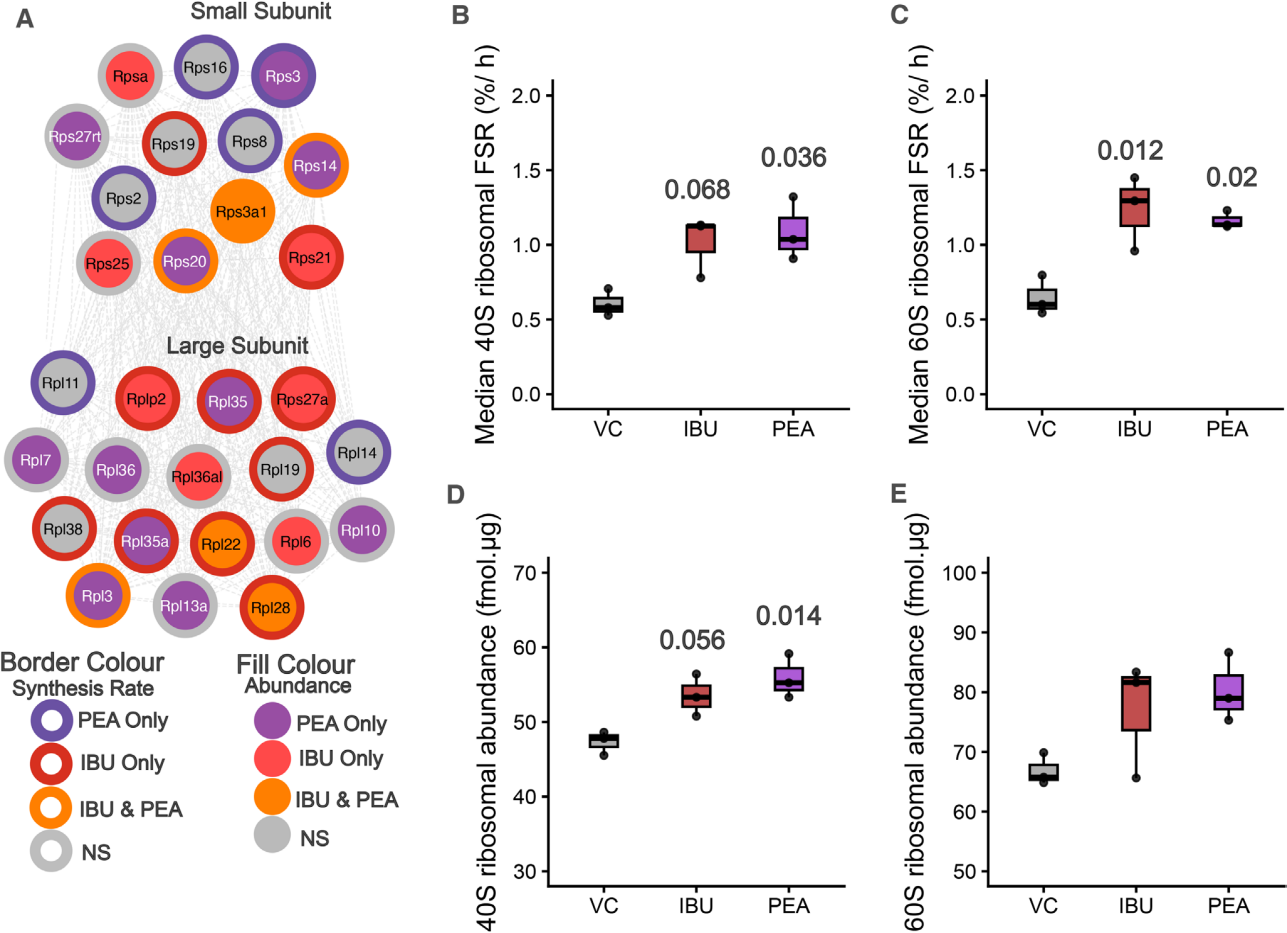
Effects of IBU and PEA treatment on ribosomal protein synthesis and abundance. (A) STRING‐based network visualization of cytosolic ribosomal proteins significantly changed in synthesis rate and/or abundance in response to different treatments. Each node represents a ribosomal protein from the large (60S) or small (40S) subunit, with border color denoting proteins significantly altered in synthesis rate and fill color denoting changes in abundance. Purple = PEA‐specific, red = IBU‐specific, orange = shared between PEA and IBU, and gray = non‐significant (NS). (B, C) Median ribosomal protein fractional synthesis rate (FSR, %/h) for the 40S (B) and 60S (C) subunits. (D, E) Ribosomal protein abundance (fmol/μg) for the 40S (D) and 60S (E) subunits. (B–E) Text annotations in panels represent Tukey‐adjusted *p*‐values for comparisons between each treatment and vehicle control‐treated C2C12 cells.

Following the observation of changes in networks of ribosomal proteins being a core component of the response to PEA and IBU, we aimed to assess changes in mixed‐ribosomal synthesis rate and total ribosome abundance following different treatments. Both PEA and IBU induced significant increases in ribosomal protein synthesis rate, across both the 40S and 60S ribosomal subunit. The 40S subunit showed a significant treatment effect (*p* = 0.032), with IBU (+67%; *p* = 0.06) and PEA (+80%; *p* = 0.04) both increased relative to control (0.61% ± 0.09%/h) (see Figure 5B). Similarly, 60S subunit synthesis rates were higher following IBU (+90%; *p* = 0.012) and PEA (+79%; *p* = 0.021) compared with control (0.65% ± 0.13%/h) (see Figure 5C). Indicating similar increases in ribosomal protein synthesis in response to both PEA and IBU.

Both treatments induced a coordinated upregulation of ribosomal protein abundance across subunits. The 40S subunit increased by +17% with ibuprofen (53.5 ± 2.83; *p* = 0.056) and + 18% with PEA (55.9 ± 2.98; *p* = 0.014) relative to control (47.3 ± 1.60) (see Figure 5D). The 60S subunit showed a comparable pattern, rising by +15% with ibuprofen (76.9 ± 9.8) and +20% with PEA (80.3 ± 5.8) versus control (66.8 ± 2.7; *p* = 0.11) (see Figure 5E). Indicating, both treatments induced concordant increases in ribosomal protein abundance and synthesis, indicating stimulation of ribosome biogenesis and enhanced ribosomal protein turnover.

### 3.6 Relationship Between Changes in Protein Synthesis and Proteome Remodeling

**FIGURE 6:**
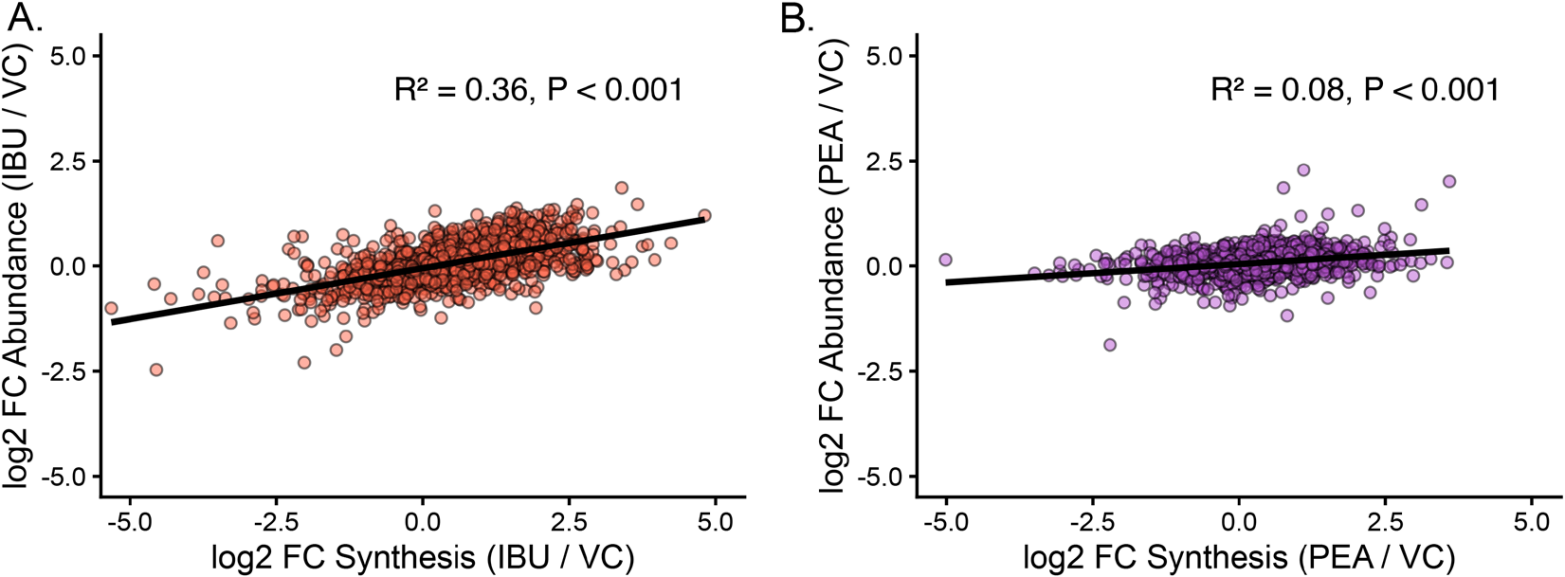
Contribution of changes in synthesis to proteome remodeling. Scatterplots show the relationship between protein‐specific changes in fractional synthesis rate (FSR; *x*‐axis) and protein abundance (ABD; *y*‐axis) following (A) ibuprofen (IBU) or (B) palmitoylethanolamide (PEA) treatment, expressed as log_2_ fold change relative to vehicle control (VC). Each point represents an individual protein detected in both datasets. Black lines denote linear regressions ±95% confidence intervals. The coefficient of determination (*R* ^2^) and associated *p* values are shown within each panel. All values represent treatment–control differences at the 36 h time point and do not reflect longitudinal within‐treatment changes.

To assess the relationship between changes in protein synthesis and abundance, correlation analyses were performed across all quantified proteins for both IBU and PEA treatments (Figure 6). For IBU, changes in FSR were moderately correlated with changes in abundance (*R* ^2^ = 0.36, *p* < 0.001), indicating that approximately one‐third of the variation in protein abundance could be explained by alterations in synthesis rate (Figure 6A). In contrast, the relationship was markedly weaker following PEA treatment (*R* ^2^ = 0.08, *p* < 0.001), suggesting a more transient co‐regulation of changes in abundance and synthesis rate (Figure 6B). Together, these findings indicate that IBU‐induced proteome remodeling more strongly reflects sustained changes in protein synthesis, whereas PEA elicits a comparatively decoupled response between synthesis and accumulation indicating a coordinated increase in protein degradation alongside synthesis resulted in relatively little protein abundance accumulation.

## 4 Discussion

This study provides the first proteome‐wide analysis of the effects of IBU and PEA on proteome dynamics in skeletal muscle cells. Using high‐resolution mass spectrometry‐based proteomics combined with deuterium oxide labeling, we resolved the abundance and synthesis rates of 3328 and 2513 proteins, respectively, across vehicle control (VC), IBU, and PEA‐treated C2C12 cells. Our findings demonstrate that both IBU and PEA enhance the synthesis and abundance of ribosomal proteins. However, IBU induced a broader spectrum of remodeling associated with increased abundance of extracellular matrix and contractile proteins alongside increased abundance of enzymes associated with lipid metabolism and concomitant decreases in the abundance of proteins associated with carbohydrate metabolism. Whereas, PEA‐induced proteome remodeling was more specific resulting in increased synthesis and abundance of predominantly only ribosomal proteins.

Previous studies have suggested that NSAIDs can suppress aspects of translational regulation and ribosomal biogenesis and are therefore, inhibitory to the acute remodeling process following exercise. For example, ibuprofen and indomethacin have been shown to inhibit insulin‐stimulated rRNA synthesis in myoblasts [55], whereas acute ibuprofen ingestion blunts post‐exercise increases in the phosphorylation of p70S6K and ribosomal protein S6 in human skeletal muscle [56]. However, these acute effects contrast with findings from prolonged NSAID administration. Recent work reports chronic administration during resistance training does not impair ribosomal biogenesis or hypertrophy [26, 57]. In our current work, IBU treatment increased both the synthesis rate and abundance of ribosomal proteins, consistent with enhanced ribosome biogenesis (see Figure 5). Ribosomal FSR increased by approximately +80%–90% relative to VC, and total ribosomal protein abundance increased by ~15%. When compared directly, both IBU and PEA produced concordant increases in ribosomal protein synthesis and abundance, indicating a shared upregulation of translational machinery in response to both treatments. Ribosomal proteome expansion has been highlighted as an early molecular feature of hypertrophic adaptation to resistance training [37, 38]. Therefore, this PEA and IBU induced increase in ribosomal proteins may suggest a potentially beneficial role in these compounds being used alongside resistance training interventions. Whilst no interference effect of NSAIDs/COX inhibitors on resistance exercise outcomes have been reported following chronic training in younger participants [39]. Studies in older participants reported greater increases in muscle volume and strength in the resistance training plus NSAID group [12]. Follow up analysis revealed COX inhibitor consumption during resistance exercise in older individuals enhanced myocellular growth, particularly in Type I muscle fibers [40]. Therefore, these data suggest under circumstances where sufficient muscle protein synthetic responses occur (e.g., in young, healthy individuals following resistance exercise), there may be no further benefit of IBU/PEA supplementation combined with training. However, the aging‐specific potentiation of resistance training outcomes may suggest that in contexts where there is impaired protein turnover, there may be a beneficial effect of these treatments as an adjunct supplement to training. This raises the possibility that modulation of ribosome abundance and turnover via administration of IBU or PEA may facilitate recovery and adaptation, particularly in the context of exercise‐induced muscle damage or recovery or where basal protein turnover is impaired. Interestingly, although both compounds elicited convergent increases in ribosomal protein synthesis and abundance, PEA's effects appeared more molecularly targeted, eliciting predominantly ribosomal remodeling, compared with the broader metabolic and structural remodeling induced by IBU. This distinction raises an important question as to whether PEA could represent a pharmacological means of selectively enhancing translational capacity without the wider metabolic interference associated with NSAIDs/COX inhibitors. Therefore, future studies should aim to clarify whether PEA's action on ribosomal pathways contributes to a more specific, adaptive mode of proteome regulation in muscle.

The increase in ribosomal proteins coincided with a broader proteome remodeling characterized by increased abundance of proteins associated with extracellular matrix (ECM) remodeling and muscle contraction. Although ECM remodeling during muscle regeneration and hypertrophy are similar, each processes are distinct with different satellite cell requirements [41]. Previous work investigating the effect of NSAIDs on muscle cells in vitro report high doses, similar to those used in the current work, augments myogenic cell proliferation but have no measurable effect on myotube size [26]. Therefore, highlighting NSAIDS may influence cell cycle progression and myogenic differentiation within skeletal muscle cells, rather than inducing hypertrophy of myotubes. Organization and expansion of the ECM is essential for myogenesis [42] and proteomic profiling identified the induction of the dystrophin complex, such as dystroglycan and sarcoglycan, was associated with later stage myogenesis [43]. Our current work identified significant enrichment of proteins associated with extracellular matrix proteins (e.g., Thrombospondin‐1, Nidogen‐2, Dystroglycan 1, and Cathepsins B, L, Z) increased in abundance following IBU treatment. Furthermore, the development and maturation of the contractile apparatus during myogenesis requires a parallel increase in calcium‐dependent cycling proteins to handle the rapid fluxes in Ca^2+^ associated with muscle contractility [44]. Untargeted proteomic profiling during C2C12 myoblast differentiation reported late stage differentiating cells was associated with increased abundance of calcium transporters and proteins linked to skeletal muscle excitation/contraction (such as actin, myosin, troponin, nebulin, titin, desmin, and α‐actinin) [43]. In line with these works we identified significant enrichment of calcium handling proteins to be increased in abundance following 36 h of IBU treatment. Our abundance profiling reports increased abundance of numerous proteins associated with muscle contractile apparatus, including troponins (Tnnc1, Tnnt2, and Tnnt3), Tropomyosins (Tpm1 and Tpm2), and myosin light chain proteins (Myl1, Myl4, and Myl9), as well as alpha‐actinin‐3 (Actn3). Taken together, these data indicate that IBU promotes a transition of muscle cells toward a pro‐myogenic state characterized by ribosomal expansion, ECM remodeling, and maturation of the contractile apparatus. This profile reflects activation of anabolic and structural programmes typical of differentiating or regenerating myotubes, suggesting that NSAID exposure at this stage may increase cellular processes underpinning muscle repair and adaptation.

Beyond its effects on structural remodeling, IBU treatment also induced coordinated changes in metabolic proteins, suggesting wider metabolic remodeling. In particular, IBU increased the abundance of proteins involved in lipid and fatty acid binding (e.g., fatty acid translocase CD36 and fatty acid‐binding protein 3). CD36 is a key regulator of long‐chain fatty acid uptake and oxidation in skeletal muscle, and muscle‐specific ablation of CD36 impairs exercise‐induced increases in fatty‐acid oxidation despite normal mitochondrial biogenesis [45]. Furthermore, IBU decreased the abundance of proteins associated with carbohydrate metabolism, including key glycolytic enzymes like hexokinase‐2, phosphofructokinase (PFKAP; platelet type and PFKAL; liver type) and pyruvate dehydrogenase kinase isozyme 3 (PDK3). Notably, PFKAP also plays an important role in linking macrophage activation to glycolytic control and NADPH metabolism [46, 47]. Under normal conditions, transient macrophage activation promotes glycolysis to generate NADPH that supports antioxidant defense and tissue‐repair signaling. However, chronic suppression of these pathways, such as repeated or high‐dose NSAID exposure, as applied in the current work, could attenuate glycolytic metabolism, resulting in a re‐organization of metabolic pathways toward enhanced lipid utilization. Although the present study involved acute exposure of IBU directly to muscle cells in vitro, a reduced interaction of macrophages and muscle cells might be expected in vivo where greater macrophage–muscle cross‐talk occurs. Therefore, Ibu‐induced decreases in prostaglandin‐mediated signaling may alter metabolic phenotype in vivo. Because previous NSAID‐muscle studies were largely hypothesis‐driven (e.g., focusing on ribosomal biogenesis or cell cycle status) such metabolic adaptations induced by NSAIDs have been likely overlook, which could have important implications for exercise‐induced adaptation where NSAIDs are used alongside training. Furthermore, during myogenic differentiation, muscle metabolism traditionally transitions from glycolytic to more oxidative, with mitochondrial biogenesis and fatty‐acid oxidation pathways up‐regulated [48]. This metabolic switch further supports structural protein responses, highlighting a change in cell status toward a more mature, oxidative, contractile phenotype. Taken together, the metabolic signature we observe aligns with the pro‐myogenic programme evidenced by ECM/contractile enrichment in IBU treated muscle cells reinforcing the concept that IBU promotes a shift in cell cycle characteristics toward that of later stage myogenic differentiation.

The relationship between protein synthesis rate and protein abundance differed between IBU and PEA when evaluated relative to vehicle control (VC). In response to IBU treatment, there was a moderate positive correlation between the treatment–control differences in FSR and the corresponding differences in protein abundance (*R* ^2^ = 0.36; Figure 6A). This indicates that proteins exhibiting higher synthesis rates relative to VC also tended to display greater abundance relative to VC at the 36 h time point. Such concordance between treatment‐induced differences in synthesis and abundance is consistent with an anabolic profile of proteome remodeling, comparable to that observed following resistance exercise [22, 38], where elevated synthesis drives net accretion of structural and contractile proteins. In contrast, the relationship between treatment–control differences in synthesis and abundance following PEA exposure were weak (*R* ^2^ = 0.08; Figure 6B), despite 101 proteins exhibiting elevated synthesis rates relative to VC. Although 75 proteins showed nominally significant abundance differences (*p* ≤ 0.05), all corresponding q‐values were 0.99, indicating no statistically robust differences in abundance between PEA and VC at 36 h. Importantly, these analyses compare PEA directly to VC and do not assess temporal changes within PEA‐treated cells. Therefore, although PEA increased synthesis rates relative to control, we did not detect corresponding differences in steady‐state abundance relative to VC within the 36 h exposure period. This pattern suggests that PEA‐induced increases in synthesis may not translate into detectable treatment–control differences in protein abundance over this time frame. One possible interpretation is that PEA elevates protein turnover, whereby increased synthesis is matched by proportional degradation, thereby maintaining steady‐state abundance relative to control. However, direct measures of degradation would be required to confirm this mechanism. Increased turnover is a key component of muscle protein homeostasis and is recognized as essential for maintaining protein quality in skeletal muscle [29, 49, 50]. PEA may therefore facilitate improvements in proteome quality control within muscle, thus aiding recovery processes following acute exercise or damage through increased degradation and recycling of damaged/dysfunctional proteins. Collectively, these data suggest PEA enhances turnover, which in turn may lead to improvement in muscle proteostasis.

In the present study, no significant differences were observed in mixed‐protein FSR between treatments (see Figure 2A). However, such measures represent the average synthesis of thousands of proteins, each of which may respond differently to a given intervention. As a result, bulk mixed‐protein estimates can mask important, protein‐specific effects. Consistent with this, whereas global FSR appeared similar between treatments, IBU significantly altered the synthesis of 172 proteins and PEA altered 103 proteins relative to vehicle control (Figures 3 and 4). Moreover, the moderate correlation between protein‐specific responses to IBU and PEA (Figure 2B) suggests both shared and treatment‐specific regulation of synthetic processes. These findings underscore the value of dynamic proteome profiling in revealing selective pharmacological effects on muscle protein metabolism that are invisible to conventional mixed‐protein or abundance‐based approaches. By resolving individual protein turnover rates, this approach enables identification of subtle yet coordinated shifts in synthesis within distinct molecular networks, providing mechanistic insight into how pharmacological agents, such as NSAIDs or lipid amides, influence the muscle proteome beyond their classical anti‐inflammatory actions.

Several limitations should be acknowledged. First, the use of an in vitro C2C12 model allows for the controlled investigation of cellular responses, but it does not capture the systemic complexity of muscle tissue in vivo, including immune, vascular, and neural interactions that may influence drug responses. Moreover, C2C12 cultures contain a subpopulation of unfused myoblasts as identified in previous studies [51], which therefore may introduce variability in protein expression patterns.

This study provides novel mechanistic insight into the direct actions of PEA and IBU on skeletal muscle, but the pharmacokinetics and metabolism of PEA and IBU in vivo are not replicated in cell culture studies. IBU undergoes extensive hepatic metabolism, primarily in the liver, via cytochrome P450 enzymes (mainly CYP2C9) and subsequent glucuronidation, which influences its half‐life, tissue distribution, and local concentration [52]. PEA, on the other hand, is primarily metabolized locally within tissues, including the liver, intestine, immune cells, and skeletal muscle, by the hydrolytic enzymes fatty acid amide hydrolase (FAAH) and N‐acylethanolamine‐hydrolysing acid amidase (NAAA) [2]. Importantly, the relative contribution of FAAH versus NAAA to PEA metabolism is tissue‐dependent and may vary with cell type, pathological state, or whether PEA is endogenously produced or exogenously applied [2]. These enzyme expression patterns and individual variability introduce significant complexity when translating in vitro results to in vivo, as such, the effects observed in this C2C12 model may differ under physiological conditions where PEA metabolism and distribution are regulated.

Finally, the concentrations used in this study were selected based on prior dose–response experiments and existing literature [26]. One key issue is the use of supratherapeutic concentrations of IBU in vitro. A review by Graham and Scott [53] highlights that many of the cellular effects attributed to NSAIDs, including IBU, are observed only at concentrations that far exceed those attainable in vivo. This is particularly important when considering the high degree of plasma protein binding that limits the free, pharmacologically active fraction of IBU in circulation [54]. As a result, in vitro studies using micromolar or even millimolar concentrations may not reflect the true biological activity of IBU under physiological conditions. These discrepancies raise important concerns regarding the relevance and translatability of such findings to in vivo contexts, where drug metabolism, distribution, and systemic interactions are tightly regulated.

## 5 Conclusion

Acute treatment with IBU and PEA induced largely distinct dynamic proteomic responses in C2C12 myotubes. Interestingly, in response to both treatments we observed a conserved increase in ribosomal protein synthesis and abundance, consistent with enhanced translational capacity in muscle. However, IBU exerted broader effects on protein‐specific remodeling increasing networks of proteins associated with a pro‐myogenic phenotype. Potentially highlighting the ability of IBU to support adaptation and regeneration following exercise and injury. Whereas the response to PEA was mostly specific to only changes in ribosomal protein abundance and induced increases in global rates of protein turnover. Therefore, PEA may offer the potential to improve muscle protein homeostasis and quality control pathways without overt proteome reorganization. These findings demonstrate the value of dynamic proteomics in resolving mechanistic differences between interventions that are not apparent from bulk/static measures alone and provide mechanistic insight into how IBU and PEA may influence muscle adaptation and support recovery to acute exercise or injury.

## Author Contributions

P.L.C. performed experiments, performed analysis, and was involved in manuscript preparation and review; C.A.S. performed mass spectrometry experiments, performed bioinformatics analysis, and was involved in manuscript preparation and review; J.G.B. and D.J.O. conceived the experimental idea and plan, performed analysis, and were involved in manuscript preparation and review.

## Funding

Gencor Pacific Ltd.

## Conflicts of Interest

The authors declare no conflicts of interest.

## Supporting information


**Figure S1:** STRING protein–protein interaction networks of significantly regulated proteins (two‐way ANOVA interaction effect, *p* < 0.05; *n* = 418) clustered into five groups (*k* = 5) across treatment conditions (VC, ibuprofen, and PEA).

**Data S1:** Statistical outputs of 2 way ANOVA of abundance data.

**Data S2:** Raw data.

**Data S3:** Statistical outputs.

## Data Availability

The mass spectrometry proteomics data have been deposited to the ProteomeXchange Consortium via the PRIDE [28] partner repository with the dataset identifier PXD069882 and http://doi.org/10.6019/PXD069882. Processed and analysis‐ready datasets, along with the full R analysis pipelines used to generate all figures and statistical outputs, are available via GitHub at: https://github.com/cstead27/C2C12_PEA_IBU_Dynamic_Proteomics.
