# Supplementary material for: The Effects of Palmitoylethanolamide or Ibuprofen on the Abundance Profile and Synthesis Rate of Proteins in C2C12 Skeletal Myotubes
Source: FASEB Bioadv. 2026 Apr 23;8(4):e70108. doi: 10.1096/fba.2025-00286 (PMC13104729; doi:10.1096/fba.2025-00286)
Supplement: Supplementary file 1 — Figure S1: STRING protein–protein interaction networks of significantly regulated proteins (two‐way ANOVA interaction effect, p < 0.05; n = 418) clustered into five groups (k = 5) across treatment conditions (VC, ibuprofen, and PEA). [file FBA2-8-e70108-s001.html]

Interaction Clusters · ABD Analysis


# Interaction Clusters  ·  STRING Networks

2-way ANOVA interaction effect  ·  p < 0.05  ·  418 proteins  ·  k = 5 clusters  ·  VC vs Ibuprofen vs PEA

Mus musculus  ·  STRING v12  ·  score ≥ 400

Interaction effect heatmap    row z-score  ·  hierarchically clustered

−z

+z

VC

Ibuprofen

PEA

· 12h & 36h per condition
